# Vaginal Practices of HIV-Negative Zimbabwean Women

**DOI:** 10.1155/2010/387671

**Published:** 2010-08-24

**Authors:** Abigail Norris Turner, Charles S. Morrison, Marshall W. Munjoma, Precious Moyo, Tsungai Chipato, Janneke H. van de Wijgert

**Affiliations:** ^1^Division of Infectious Diseases, Department of Internal Medicine, The Ohio State University Medical Center, 410 West 10th Avenue, Doan Hall, N-11, Columbus, OH 43210, USA; ^2^Family Health International, Division of Behavioral and Biomedical Research, 2224 E NC Hwy 54, Durham, NC 27713, USA; ^3^UZ-UCSF Collaborative Research Programme, 15 Phillips Avenue, Belgravia, Harare 00263, Zimbabwe; ^4^Department of Obstetrics and Gynaecology, University of Zimbabwe, P.O. Box A178, Avondale, Harare, Zimbabwe; ^5^Academic Medical Center, University of Amsterdam, Amsterdam Institute for Global Health and Development, P.O. Box 22700, Amsterdam 1100, The Netherlands

## Abstract

*Background*. Vaginal practices (VPs) may increase HIV risk by injuring vaginal epithelium or by increasing risk of bacterial vaginosis, an established risk factor for HIV. *Methods*. HIV-negative Zimbabwean women (*n* = 2,185) participating in a prospective study on hormonal contraception and HIV risk completed an ancillary questionnaire capturing detailed VP data at quarterly followup visits for two years. *Results*. Most participants (84%) reported ever cleansing inside the vagina, and at 40% of visits women reported drying the vagina using cloth or paper. Vaginal tightening using cloth/cotton wool, lemon juice, traditional herbs/powders, or other products was reported at 4% of visits. Women with ≥15 unprotected sex acts monthly had higher odds of cleansing (adjusted odds ratio (aOR): 1.17, 95% CI: 1.04–1.32). Women with sexually transmitted infections had higher odds of tightening (aOR: 1.42, 95% CI: 1.08–1.86). *Conclusion*. Because certain vaginal practices were associated with other HIV risk factors, synergism between VPs and other risk factors should be explored.

## 1. Introduction

Vaginal practices (VPs) include douching with water, detergent, or other substances, using fingers or cloth, and insertion of natural, household, or commercially available products to cleanse, dry, or “tighten” the vagina. VPs are commonly performed by women worldwide [1–4] and are undertaken for a range of purposes: for hygiene (particularly during menstruation, prior to or following sex, or during pregnancy), for disease or pregnancy “prevention,” to meet expectations or preferences of sexual partners, or simply to follow traditional norms as learned from mothers or grandmothers in childhood [5, 6]. Importantly, VPs are frequently reported in areas of high HIV prevalence [3, 4, 7, 8]. VPs may directly increase HIV risk by causing abrasions in the vaginal epithelium or mucosal inflammation that may lead to recruitment of HIV target cells [9, 10]. VPs may also indirectly increase HIV risk by disrupting vaginal flora, leading to bacterial vaginosis (BV); BV is an established risk factor for HIV acquisition [11]. In some analyses, vaginal practices have been directly associated with increased prevalence [3, 8, 12] and incidence of HIV [13], though other studies report no significant effect of VPs on HIV risk [14, 15]. A recent meta-analysis of five longitudinal studies of African women presented a summary unadjusted effect of any kind of intravaginal practices (including cleansing, drying, and tightening) on incident HIV. The authors report “some evidence of an increased risk of HIV acquisition” due to vaginal practices, but confidence intervals were wide and heterogeneity was high (summary effect: 2.65, 95% confidence interval (CI): 0.95, 7.36, I^2^ 92.0%) [16]. 

If vaginal practices are associated with HIV risk, in-depth understanding of these behaviors is critical for the development of effective interventions aimed at changing them. Many previous studies of HIV risk which include VPs as an exposure or confounding variable have considered it a single “yes/no” variable, without further exploration of important nuances in behaviors. Using a large-scale ancillary study conducted alongside a prospective assessment of the effect of hormonal contraception on HIV risk, we aimed to evaluate the specific products, methods, and frequency of vaginal practices of HIV-negative Zimbabwean women, particularly the timing of VPs related to menses, sexual activity, and other behaviors. We wished to consider changes in vaginal practices that occurred during women's study participation and to examine whether specific VPs were significantly correlated with other reproductive health factors, including condom use, contraception, symptoms of sexually transmitted infections (STIs), and laboratory-confirmed STIs.

## 2. Materials and Methods

The hormonal contraception and the risk of HIV acquisition (HC-HIV) study was a multisite, prospective cohort study assessing the effect of hormonal contraception on HIV acquisition among women in Zimbabwe, Uganda, and Thailand. Detailed methods and results have been published previously [17]. The data presented here were collected both through HC-HIV and through an ancillary study that explored in-depth the specific vaginal practices of HIV-negative Zimbabwean women who participated in HC-HIV. 

### 2.1. Ethical Approval

All participants consented to the research. The project received ethical review and approval by the Protection of Human Subjects Committee at Family Health International (Durham, NC) and the Medical Research Council of Zimbabwe.

### 2.2. Study Setting and Population

These analyses are restricted to Zimbabwean participants in the HC-HIV study who participated in the ancillary VP study. HC-HIV recruited women from 1999–2002. Eligible women were 18–35 years of age, HIV-seronegative, sexually active (≥ three acts in the past three months), and using either combined oral contraceptive pills (COCs), injectable depot medroxyprogesterone acetate (DMPA), or a nonhormonal or no contraceptive method. All Zimbabwean participants were recruited from family planning and maternal-child health clinics.

### 2.3. Data Collection

At enrollment and each followup visit, women in HC-HIV received structured interviews about their reproductive, contraceptive, and sexual behavior and physical exams with specimen collection. Visits were conducted approximately every 12 weeks. 

VPs were measured through two mechanisms. First, HC-HIV participants were asked quarterly, “Since your last regular visit, did you ever use anything to dry or tighten your vagina for sex?” and “Since your last regular visit, did you use anything to clean the inside of your vagina, for instance, when you were bathing?” If women answered affirmatively, they were asked followup questions about the frequency of these behaviors and the products they used. Second, as part of the VP ancillary study, participants completed an additional four-page, interviewer-administered, structured survey that explored VPs in greater detail, including timing related to contraception, sex, and menstruation, and the precise form and dose of commercial or traditional cleansing, drying, or tightening products (survey available upon request). Importantly, the ancillary study asked questions about drying separately from tightening; these concepts were combined in the HC-HIV questionnaire. The following analyses use both data sources.

### 2.4. Statistical Analyses

Statistical analyses were performed using SAS (Version 9.2, SAS Institute, Cary, NC). 

We restricted the analysis to Zimbabwean women who completed at least one followup visit with valid HIV results and provided information about their vaginal practices on the ancillary study questionnaire during at least one followup visit. We excluded women who did not return to the clinic after enrollment or who never completed the VP ancillary study questionnaire. 

Because of HC-HIV's primary aim, women were censored at the visit at which they were found to be HIV-infected. Women were considered HIV-infected if positive on a combination of two enzyme-linked immunosorbent assays (ELISA) or rapid tests. Positive results were confirmed by Western Blot or HIV polymerase chain reaction (PCR).

At each visit, clinicians collected a single endocervical swab for PCR identification of both gonococcal and chlamydial infection (AMPLICOR Ct/NG Test, Roche Diagnostics, Somerville, NJ, USA). Trichomonal infection was diagnosed using wet mount with examination under low (10x) and high (40x) magnification. Presence of motile flagellated trichomonads indicated trichomoniasis. Bacterial vaginosis was diagnosed through gram-stained vaginal smears using Nugent scoring [18].

We examined univariate and bivariate frequencies of vaginal practices reported by women both during their HC-HIV quarterly visits and on the VP ancillary study questionnaire. We also ran unadjusted and adjusted multivariable logistic regression models to estimate the associations between various reproductive health factors (frequency of unprotected sex, contraceptive use, STI symptoms in the last three months, and diagnosed chlamydial, gonococcal, or trichomonal infection at the current visit) and reported vaginal practices [19]. We modeled vaginal cleansing, drying, and tightening as separate outcomes of interest. We used generalized estimating equations (GEE) to account for clustering from repeated visits by individual women [20, 21]. For each of the three models, we adjusted for the reproductive health factors above as well as other fixed and time-varying variables that we hypothesized may be associated with vaginal practices, including age, years of education, cohabitation status, primary partner's circumcision status, and sexual frequency in a typical month in the last three months. We did not examine associations between vaginal practices and incident HIV; a previous analysis of the HC-HIV data reported an adjusted hazard ratio of 0.81 for HIV acquisition (95% confidence interval, 0.59 to 1.10) for women who reported any vaginal practice in the last three months (including vaginal drying, tightening, or cleansing) [15].

## 3. Results

The analysis dataset was comprised of 2,185 women, 95% of the 2,296 Zimbabwean women enrolled in the parent HC-HIV study. Longitudinal analyses included 14,154 followup visits and 3,569 person-years (PYs) of followup time. The median number of visits per participant was 8 (range: 2 to 11) and median followup time per participant was 21.4 months (range: 1.9 to 42.7 months). 

Overall, participants' median age at baseline was 26 years (interquartile range (IQR): 22 to 29 years), and they had a median of 11 years of education (IQR: 9–11) (Table 1). Engagement in commercial sex was rare (1% of participants at baseline). Women's median sexual frequency was 14 acts per month (IQR: 8–22 acts), and the median number of sex partners in the past three months was 1 (IQR: 1-1 partners). Almost all participants (93%) were cohabitating with a husband or a boyfriend (Table 1). 

Two-thirds of participants (66%) reported any VP at baseline, including vaginal cleansing, drying, or tightening. We observed few demographic differences between women engaging in VPs and women who did not at baseline (Table 1): cohabitation status, history of commercial sex, age, education, and sexual behaviors were similar between the two groups. Women who reported no VPs in the previous three months were somewhat more likely to be users of combined oral contraceptives (41% versus 36% of women who did report VPs at baseline). 

In the quarterly HC-HIV interviews, 1917 women (88% of participants) reported at 10,402 followup visits (74% of visits) that they ever cleaned inside the vagina in the last three months. Participants reported cleansing a median of 60 times per month (IQR: 30–60 times), the equivalent of roughly twice per day. Women reported at fewer visits (*n* = 3,178, 22% of visits) that they had used something to dry or tighten the vagina; 924 women (42% of participants) ever gave this response during followup. The frequency of drying/tightening (among women reporting this behavior) was also less than for cleansing: among those participants reporting drying or tightening, women dried or tightened the vagina a median of 20 times in the last month (IQR: 6–30 times), less than once per day on average. 

Agreement on reported vaginal cleansing between the quarterly HC-HIV surveys and the VP ancillary study was good. In response to the general question for the ancillary study, “Do you wash inside your vagina?” 1,933 women (88% of participants) reported cleansing inside the vagina at 10,598 followup visits (75% of all followup visits). The majority had last cleansed on the same day as the visit (89%) or one day previously (4%). Products used for cleansing included plain water only (86% of visits where cleansing was reported) and water with soap (13% of visits). Women reported using disinfectants such as Dettol (a liquid, phenol-based household antiseptic product) or Betadine at 66 visits (<1% of visits where cleansing was reported). Cleansing with salt solution, vinegar, or lemon juice was very rare, reported at 18, 3, and 2 visits, respectively. 

The VP ancillary questionnaire next asked women about products they may have inserted into the vagina during menstruation in a typical month. Thirty-three percent of participants (*n* = 723) ever reported inserting products during menstruation during the followup period. Insertion of products during menstruation was reported at 2,026 followup visits (14%), with the majority inserting “cotton wool” (absorbent cotton like that in a pill bottle), tampons, or cloth. 

The ancillary study also explored practices related to vaginal drying. At 41% of followup visits, 1,459 individual women (67% of participants) reported that they used a towel, cloth, paper or, cotton wool to dry the vagina. At more than half of these visits (57%), the last time women dried the vagina was the same day as the visit, with an additional 17% last drying the vagina during the previous day. Most women reported moving their fingers in their vagina with the drying product (63% of visits where vaginal drying was reported). The median duration of time that the drying product was left in the vagina after insertion was 3 minutes (IQR: 1–30 minutes). 

The ancillary study survey further questioned women about products they may have inserted to tighten the vagina. Overall, 12% of women (*n* = 272) ever answered “yes” to the general question, “Do you insert anything to tighten your vagina?” during followup. Vaginal tightening was reported at 574 visits (4% of followup visits). Most women who reported inserting products for vaginal tightening had last done so recently: 20% on the same day as the visit, 20% the day before the visit, and 34% between 2 and 7 days before the visit. The median time that tightening products were left in the vagina was 75 minutes (IQR: 30–180 minutes). Women reported at 20 visits (3% of visits where tightening was reported) that they left the product in place during sex. The specific products which women inserted to tighten the vagina were extremely varied (Table 2). The most common included cloth (28% of visits where vaginal tightening was reported), lemon or lemon juice (21%), cotton wool (20%), natural substances including herbs, powders, and roots (16%), water (14%), and salt or salt solution (7%) (Table 2). Most products were used independently rather than in combination. However, cloth and/or cotton wool were sometimes used in conjunction with lemon/lemon juice (38 of 120 reports of lemon juice also included use of cloth or cotton wool), salt/salt solution (12 of 40 reports of salt/salt solution also included cloth or cotton wool), and oil (5 of 14 reports of oil also included use of cloth or cotton wool).

**Figure 1:**
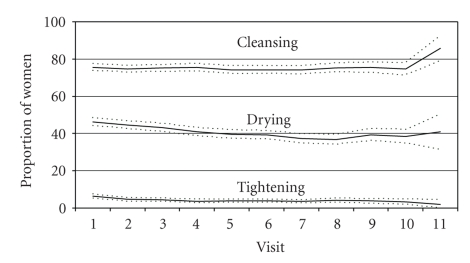
Proportion and 95% confidence intervals of reported vaginal cleansing, drying, and tightening, over study duration.

**Figure 2:**
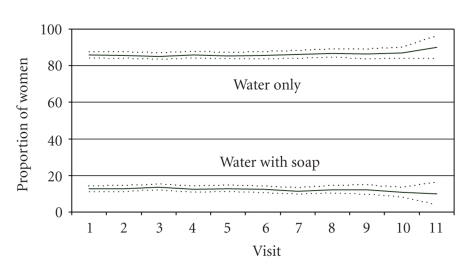
Proportion and 95% confidence intervals of reported vaginal cleansing with water only versus water and soap, among women reporting vaginal cleansing, over study duration.

We next explored whether women's vaginal practices changed over time (Figure 1). Cleansing, drying, and tightening behaviors reported during the VP ancillary study were fairly constant over the followup period, with the proportion reporting cleansing hovering around 75%, the proportion reporting drying fluctuating around 40%, and the proportion reporting tightening remaining around 4% (Figure 1). Among those reporting cleansing, the proportion that used water only and the proportion that used water with soap was also remarkably consistent over time (Figure 2).

Finally, we evaluated whether reported vaginal cleansing, drying, or tightening were associated with (a) the number of unprotected sex acts (i.e., sex acts where condoms were not used) in a typical month in the last three months; (b) current contraceptive use (nonhormonal methods, COCs or DMPA); (c) STI symptoms (a composite variable capturing any vaginal discharge, genital itching, abdominal pain, pain during sex, and/or irregular bleeding in the last three months); or (d) diagnosed STI (laboratory-confirmed chlamydial, gonococcal, or trichomonal infection) (Table 3). 

In unadjusted analyses, women who reported at least 15 unprotected acts in a typical month in the last three months, compared to those reporting fewer than five unprotected acts, had higher odds of reporting vaginal cleansing (OR: 1.17, 95% CI: 1.07, 1.29) and vaginal drying (OR: 1.10, 95% CI 1.00, 1.20). The number of unprotected acts was not associated with reported vaginal tightening. COC users, compared to users of nonhormonal contraceptive methods, had higher odds of vaginal cleansing (OR: 1.22, 95% CI: 1.07, 1.40), though contraceptive group was not significantly associated with odds of drying or tightening. Women who had STI symptoms in the last three months had significantly higher odds of reporting vaginal cleansing (OR: 1.13, 95% CI: 1.04, 1.23) and drying (OR: 1.09, 95% CI: 1.00, 1.19), but not tightening. Finally, women with laboratory-confirmed chlamydial, gonococcal, or trichomonal infection did not have higher odds of vaginal cleansing or drying, but they did have increased odds of reporting vaginal tightening (OR: 1.42, 95% CI: 1.07, 1.88). Aside from slight reductions in precision, estimates changed very little following adjustment for fixed and time-varying covariables including age, years of education, cohabitation status, partner circumcision status, contraception, sexual frequency in a typical month in the last three months, STI symptoms in the last three months, and diagnosed STI at the current visit (Table 3).

## 4. Discussion

Nearly 9 out of every 10 women in this sample of HIV-negative Zimbabwean women reported vaginal cleansing; about two-thirds reported vaginal drying and a smaller proportion, 12%, reported inserting products to tighten the vagina. These frequencies are similar to behaviors reported in a cross-sectional evaluation of a cohort of South African women participating in a randomized trial for cervical cancer screening [8]. In that study, all women reported some type of intravaginal practice, with 86% of women inserting water and much lower proportions inserting soap (18%), household antiseptics (12%), industrial detergents (5%), vinegar (4%), and salt water (2%) [8]. Vaginal practices in a prospective cohort of Kenyan sex workers were also frequent, with 71% reporting using soap or other detergent/antiseptic substances for vaginal cleansing at baseline and 23% reporting vaginal cleansing with water only [13]. In the Methods for Improving Reproductive Health in Africa (MIRA) HIV prevention trial, vaginal practices were commonly reported at baseline by general population women in Zimbabwe and South Africa; at enrollment, 83% of participants reported vaginal washing, 56% reported wiping out the vagina, and 21% reported inserting dry or absorbent materials into the vagina [22].

Reported vaginal practices were remarkably consistent over the followup period. At each visit, clinicians briefly counseled women against using abrasive drying and tightening agents like powders and herbs; some also instructed women not to cleanse inside the vagina using their fingers. Given the stability of reported behaviors, clinician counseling apparently had little impact on women's decisions regarding vaginal practices. Several recent candidate vaginal microbicide trials instructed participants to abstain from cleansing and other vaginal practices during trial participation. Whether women heeded this advice more frequently in those trials compared to this study is not known, but frequent vaginal practices such as those reported in this population could affect microbicide retention in the vagina and consequent disease-prevention efficacy.

The adjusted results from multivariable models indicate that women with a higher absolute number of unprotected acts had higher odds of engaging in vaginal cleansing. In addition, those with recent STI symptoms had higher odds of both cleansing and drying. These associations are in the expected direction. Women with the highest sexual frequency may feel more pressure to be “clean” for themselves and their partners, and the presence of seminal fluid following sex may lead them to cleanse more than women who have sex less often. If they believe that cleansing provides protection from disease acquisition, they may also cleanse as a preemptive prevention measure or “cure”. Additionally, women with STI symptoms (including bleeding, discharge, itching, and pain) may be more likely to engage in more frequent vaginal cleansing and drying to relieve discomfort. The associations between STI symptoms and both vaginal cleansing and vaginal drying persisted after adjustment for contraceptive use and other variables, suggesting that the increased vaginal bleeding sometimes associated with DMPA did not confound these associations. The finding that women with laboratory-diagnosed STI had higher odds of vaginal tightening (but not cleansing or drying) bears further study, particularly in the very small group of women who reported leaving tightening products in the vagina during sex. The temporal association of these variables—that is, whether tightening increased susceptibility to STI, or whether prevalent STI led women to engage in tightening practices—is not known because both of these measures were collected at the same time point. 

Women in our trial who reported inserting products to tighten the vagina used a range of kitchen, household, and traditional substances, including cotton wool, lemon juice, cloth, herbs, powder, roots, salt, disinfectant, oil, Vicks, sugar, vinegar, baking soda, ice, and toothpaste. These findings agree with several previous studies which investigated the prevalence and type of vaginal practices by women worldwide [23–29]. One of the most recent and comprehensive was a large qualitative study that conducted individual interviews with men and women in Indonesia, Mozambique, South Africa, and Thailand [5]. Like the practices of participants in our analyses, women in these countries also reported using a range of substances for various cleansing, drying, and tightening functions, including traditional formulations of herbs, leaves, and bark, food ingredients (lemon juice, vinegar), and commercially available products such as douching solutions, soaps and detergents, and vaginal creams [5]. 

The primary limitation of these analyses is that our vaginal practices assessment, like most measurements in sexual health, is self-reported and subject to courtesy and recall biases. In addition, as mentioned above, although the data were collected longitudinally, the interval between visits was long enough that it was not possible in multivariable models to assess the temporal association between various reproductive health factors and vaginal practices; for example, it is not clear from our analysis whether STI symptoms preceded or followed vaginal drying. As such, our measures of effect should be interpreted as correlations only and without an inference of causality. While these limitations must be noted, we believe our analyses make an important contribution to the literature on vaginal practices among African women due to the large size of our study, the comprehensive set of measures we captured on many aspects of vaginal practices, and the ability to adjust for many potential confounding factors collected as part of the parent HC-HIV study.

## 5. Conclusions

In this Zimbabwean population, vaginal cleansing was commonly reported, whereas vaginal drying was less frequent and vaginal tightening was rare. Because certain vaginal practices were associated with other HIV risk factors, synergism between VPs and other risk factors for HIV/STI should be explored. 

## Figures and Tables

**Table 1:** Selected baseline demographic characteristics of participants (*n* = 2185), stratified by vaginal practices reported at baseline.

	Any reported VP^∗,†^ in last 3 months		No reported VP in last 3 months		Overall	
Participant characteristics	*n* = 1435	%	*n* = 749	%	*n* = 2185^‡^	%

Contraceptive group						
COC*	511	36	306	41	817	37
DMPA*	522	36	236	32	758	35
Nonhormonal	402	28	207	28	610	28
Cohabitating with primary partner						
Yes	1328	93	697	93	2026	93
No	107	7	52	7	159	7
Ever engaged in commercial sex						
Yes	15	1	6	1	21	1
No	1420	99	743	99	2164	99

	Median	IQR	Median	IQR	Median	IQR

Age (years)	26	23–29	25	22–28	26	22–29
Schooling (years)	11	9–11	11	9-11	11	9–11
Nights partner away in last month	0	0–2	0	0–2	0	0–2
Sex acts with primary partner per month	14	8–24	14	8–20	14	8–22
Partners in last 3 months	1	1-1	1	1-1	1	1-1

*COC = combined oral contraceptives; DMPA = depot medroxyprogesterone acetate; IQR = interquartile range; VP = vaginal practices.

^†^Includes reports of internal vaginal cleansing, drying, or tightening.

^‡^One participant was missing vaginal practices data at baseline, thus one additional person is included in the “overall” column.

**Table 2:** Products inserted by women to tighten the vagina.

Products inserted by women	Visits (*n* = 574)	
	*n*	%*
Cloth	161	28
Lemon or lemon juice	120	21
Cotton wool	117	20
Natural substances (herbs, powder, roots)	93	16
Water	77	14
Salt or salt solution	40	7
Disinfectant (Dettol, Betadine)	16	3
Oil	14	3
Alum powder	10	2
Newspaper	6	1
Tissue paper	4	1
Powder (unspecified type)	3	1
Vicks	3	1
Sugar	2	<1
Vinegar	2	<1
Baking soda	1	<1
Ice	1	<1
Toothpaste	1	<1

*Categories sum to >100% because multiple responses were permitted.

**Table 3:** Adjusted and unadjusted measures of effect between vaginal cleansing, drying, and tightening and reproductive health factors.

Reproductive health factors	Vaginal cleansing				Vaginal drying				Vaginal tightening			
	Unadjusted		Adjusted*		Unadjusted		Adjusted*		Unadjusted		Adjusted*	
	OR^†^	95% CI^†^	OR	95% CI	OR	95% CI	OR	95% CI	OR	95% CI	OR	95% CI
Number of unprotected acts in a typical month in the last 3 months												
<5 unprotected acts (referent)	1.	—	1.	—	1.	—	1.	—	1.	—	1.	—
5–14 unprotected acts	1.06	0.98, 1.15	1.04	0.95, 1.13	1.06	0.98, 1.14	1.07	0.99, 1.16	0.84	0.69, 1.03	0.86	0.70, 1.07
15+ unprotected acts	1.17	1.07, 1.29	1.17	1.04, 1.32	1.10	1.00, 1.20	1.03	0.93, 1.15	1.04	0.83, 1.30	1.00	0.76, 1.32

Current contraceptive group												
Nonhormonal users (referent)	1.	—	1.	—	1.	—	1.	—	1.	—	1.	—
COC^†^	1.22	1.07, 1.40	1.17	1.00, 1.35	1.00	0.88, 1.13	0.88	0.77, 1.00	1.00	0.74, 1.34	0.95	0.70, 1.30
DMPA^†^	1.14	0.98, 1.33	1.10	0.93, 1.29	1.03	0.90, 1.17	0.93	0.81, 1.07	0.86	0.62, 1.21	0.80	0.57, 1.14

STI^†^ symptoms^‡^ in last 3 months												
No (referent)	1.	—	1.	—	1.	—	1.	—	1.	—	1.	—
Yes	1.13	1.04, 1.23	1.10	1.01, 1.20	1.09	1.00, 1.19	1.11	1.02, 1.21	1.17	0.95, 1.44	1.12	0.91, 1.39

Diagnosed STI at current visit												
No (referent)	1.	—	1.	—	1.	—	1.	—	1.	—	1.	—
Yes	1.11	0.94, 1.33	1.04	0.87, 1.24	0.96	0.81, 1.13	0.93	0.78, 1.11	1.42	1.07, 1.88	1.42	1.08, 1.86

*Adjusted models control for the listed reproductive health factors as well as age, years of education, cohabitation status, primary partner circumcision status, and sexual frequency in a typical month in the last three months.

^†^OR = odds ratio; CI = confidence interval; COC = combined oral contraceptive; DMPA = depot medroxyprogesterone acetate; and STI = sexually transmitted infection

^‡^“STI symptoms” include any vaginal discharge, genital itching, abdominal pain, pain during sex, and/or irregular bleeding in the last three months.
